# Health Diplomacy and Global Health: Latin American perspectives

**DOI:** 10.11606/S1518-8787.2019053000936

**Published:** 2019-04-26

**Authors:** Helena Ribeiro, Deisy de Freitas Lima Ventura

**Affiliations:** I Universidade de São Paulo. Faculdade de Saúde Pública. Departamento de Saúde Ambiental. São Paulo, SP, Brasil

**Keywords:** Book Reviews, Health Diplomacy, Global Health, Resenhas de Livros, Diplomacia em Saúde, Saúde Global

## Abstract

We will analyze and comment on the book Health Diplomacy and Global Health: Latin American Perspectives, edited by Paulo Marchiori Buss and Sebastián Tobar and published by Editora Fiocruz. Throughout its 653 pages, the book brings prominent national and foreign authors in the field of Health Diplomacy and Global Health, depicting a decade in which Brazil had great international protagonism in the field of Public Health, especially in South-South cooperation, in an innovative and structuring manner. Furthermore, the chapters present theoretical aspects and basic principles of Global Health as a new field of knowledge, in which the country has been developing and sharing scientific production with a Latin American perspective, focused on the pursuit of equity and health for all peoples of the world.

## RESUMO

Analisa-se e comenta-se a obra Diplomacia em Saúde e Saúde Global: perspectivas latino-americanas, editada por Paulo Marchiori Buss e Sebastián Tobar, publicado pela Editora Fiocruz. O livro, em 653 páginas, traz contribuições de destacados autores nacionais e estrangeiros no campo da diplomacia em saúde e da saúde global, retratando uma década em que o Brasil teve grande protagonismo internacional no campo da saúde pública, sobretudo na cooperação Sul-Sul, de forma inovadora e estruturante. Ademais, os capítulos apresentam aspectos teóricos e princípios básicos da saúde global como novo campo de conhecimento, no qual o país vem desenvolvendo e compartilhando uma produção científica com uma perspectiva latino-americana, voltada para a busca da equidade e da saúde para todos os povos do mundo.

## INTRODUCTION

For more than a decade, Brazil lived an extraordinary experience of international insertion in the field of Public Health. The Brazilian State not only actively participated in the development of the emerging field of Global Health, charting in numerous international forums and earning global recognition for the excellence of some of its public health programs, but also became a reference of a critical position on the global health governance, influencing numerous countries, mainly the so-called Global South.

The characteristics and effective results of such protagonism start to be studied in depth by researchers from many countries and subjects – something that should be encouraged, as the critical understanding of this recent history is essential for future Brazilian activities. In this sense, the work launched in September 2017 by Editora Fiocruz is a fundamental contribution, with the additional quality of referring to, beyond the Brazilian case, a Latin American perspective. Health Diplomacy and Global Health: Latin American Perspectives^1^, organized by Paulo Marchiori Buss and Sebastián Tobar, fills an important gap in the Brazilian academic literature on Public/Collective Health in its international dimensions. Authored by 41 renowned national and foreign researchers, many of them of Oswaldo Cruz Foundation and some from foreign institutions, the central theme is Global Health within the international relations. Thus was born this rich experience of Health Diplomacy.

In the preface of the work, Ambassador Celso Amorim (Minister of External Relations of Brazil between 2003 and 2011, and Minister of Defense between 2011 and 2015) mentions the term “international health policy,” arena in which Brazil exercised significant protagonism both in South-South cooperation as in the promotion of regional integration – two fundamental pillars of its action, to the point of definitely housing in Rio de Janeiro (RJ) the South American Institute of Government in Health of the Union of South American Nations (ISAGS/UNASUR), intergovernmental entity of public character whose main objective is to promote the exchange, critical reflection knowledge management and the generation of innovations in the field of Health policies and governance.

Authors who actively participated in the health diplomacy exerted by the Brazilian State in the last decades are present in the book, as is the case of the work organizers. The book systematizes a collection built by researchers from Fiocruz, institution whose excellence has greatly qualified the Brazilian international action and, moreover, was a pioneer in academic production in the emerging field of Global Health. There are contributions, beyond that of the organizers, of Celia Almeida, Ligia Giovanella, José Paranaguá de Santana, Roberto Ferreira and Luiz Eduardo Fonseca, among other renowned researchers from Fiocruz. Nonetheless, the work surpasses this collection, giving voice to authors from other institutions that are international references in the field of Global Health, such as Ilona Kickbusch, Oscar Feo, Ronald Labonté, Mario Dal Poz and Mauricio Lima Barreto, among other prominent guest writers.

Structured into five parts, the book poses valuable conceptual considerations in a field marked by the polysemy of its basic categories (Part I); identifies contemporary challenges in Global Health and Health Diplomacy (Part II); focuses on health and development from the perspective of governance (Part III); analyzes the cooperation and diplomacy in Health in Brazil (Part IV), including a chapter on Health and Health Diplomacy in the BRICS; and deals with some applications of Health Diplomacy (Part V).

Chapter 5, Health Cooperation in a Bioethics Perspective, by José Paranaguá Santana and Volnei Garrafa, illustrates the originality and appropriateness of this work. They report a bibliographical research in which they sought to identify the literature on the theme using the search term “health diplomacy,” which result in only three articles in BVS/Bireme and 114 in PubMed/NCBI, in 2011.

Additionally, the book brings about relevant data, which allow the reader to understand international iniquities in Health and the opportunity of structuring South-South cooperation. The table on page 550, comparing key indicators of the BRICS countries, highlights data showing the enormous differences between countries. For instance: the maternal mortality rate (per 100,000 live births, 2010) ranged from 34 in Russia, passing by 56 in Brazil and 200 in India, culminating to 300 in South Africa. The mortality of children under five years old per 1,000 live births also varied: 10.3 in Russia, 14.4 in Brazil, and 44.6 in South Africa.

The work emphasizes Health in Regional Integration Processes of Latin America and Caribbean (chapter 13), to reduce risks of epidemics and other problems that surpass borders and to take advantage of opportunities in the field of Health. Among such opportunities are the formation of a regional bloc to face problems such as tobacco control and abusive consumption of alcohol, as well as the production, trafficking and consumption of drugs. Likewise, the dialogue and negotiations with the medical-industrial-financial complex (private corporations that profit from disease) can happen from the integration of countries in the region, to advance in the construction of well-being of their peoples. Therefore, integration processes are one of the driving axles of external policy of many Latin American countries.

In addition, the book flaps feature comments by José Gomes Temporão, who held the functions of Minister of Health of Brazil and Director of ISAGS in a golden period for international cooperation in Health. Temporão highlights “the clear importance of Health as an international issue.” Besides its indispensable didactic material, the content of the work commented here has the potential to spark a deep reflection, one that is able to avoid setbacks as to the recognition of this importance highlighted by Temporão.

According to the organizers, they made an option for connection in the very title of the book the terms Global Health – which concerns the dimension of Health as a local product, made from conceptual and practices of multiple actors involved, in a supranational dimension – and Health Diplomacy, which is the conceptual and practical field of relations between governments and nations (also including the civil society), also socially constructed, with Health as its object (Buss and Tobar, 2017, p. 24).

In the current economic and social circumstances of Latin America, a book which seeks to explain Health on the global stage and to shape it socially and economically is useful for the training of teachers, researchers, and students of Public Health. Equally important is to understand how the global dialogues with the local, and how the Global Health Governance happens.

The factor that motivated this work was the international complexity of recent decades, coupled with the current conjuncture. Deep social and health inequalities between countries and within them were accentuated by the environmental compromise on a planetary scale, by economic and humanitarian crises related to armed conflicts, and by the mass migrations of those who are excluded and socially unprotected.

The book presents some conceptual chapters, important for the establishment of this new field of research, knowledge and expertise. In them, some basic and ethical principles of Global Health in its Latin American strand are reinforced, namely: the importance of South-South cooperation between countries facing similar problems and that can exchange innovative solutions, respecting the national sovereignty, independence and equity, without impositions and seeking mutual benefits. Buss and Ferreira advocate “structuring cooperation in health,” which implies in strengthening structuring institutions of national health systems so that they can take on an agenda of development for the future, incorporating social and environmental determinants of health and intersectoral actions.

The work also discusses the current challenges of health systems and the Sustainable Development Goals in its relations with Health. Another point to be emphasized is the presentation of various agreements, guidance documents, with their primordial content, which, on the one hand, make some chapters boring but, on the other, serve as a rich collection for those who seek references and who want to delve into the subject.

As an organized work, with a large number of authors, it is almost natural that few repetitions occur as they can be arguments for different approaches. Without harming readability, they point to the interconnection of subjects dealt with and a certain harmony of thought of the authors.

As a literary production to be used in courses on Health Diplomacy and Global Health, it falls right in the primordial goal of Academic Global Health, that is, to improve the life of all peoples around the world^2^. It is also in line with the Academic Global Health in epistemological terms, not in the sense of being a subject but a inter- or trans-subject that seeks the integration and implementation of more accessible technologies, including social ones. Similarly, the reading of the book allows one to identify some principles of Academic Global Health, namely: respect for human rights and the dignity of peoples, the pursuit of equity, ethics and sustainability.
